# Neopterin and procalcitonin are suitable biomarkers for exclusion of severe *Plasmodium falciparum *disease at the initial clinical assessment of travellers with imported malaria

**DOI:** 10.1186/1475-2875-9-255

**Published:** 2010-09-14

**Authors:** René te Witt, Marlies E van Wolfswinkel, Pieter L Petit, Jaap J van Hellemond, Rob Koelewijn, Alex van Belkum, Perry JJ van Genderen

**Affiliations:** 1 Erasmus Medical Centre, Department of Medical Microbiology and Infectious Diseases, 's Gravendijkwal 230, 3015 CE, Rotterdam, The Netherlands; 2 Harbour Hospital and Institute for Tropical Diseases, Department of Internal Medicine, Rotterdam, The Netherlands; 3 Vlietland Hospital, Department of Medical Microbiology, Schiedam, The Netherlands

## Abstract

**Background:**

Most clinicians in developed, non-malaria endemic countries have limited or no experience in making clinical assessments of malaria disease severity and subsequent decisions regarding the need for parenteral therapy or high-level monitoring in febrile patients with imported malaria. In the present study, the diagnostic accuracy of plasma soluble Triggering Receptor Expressed on Myeloid cells 1 (TREM-1), neopterin and procalcitonin levels as biomarkers for severe *Plasmodium falciparum *disease was evaluated in 104 travellers with imported malaria (26 patients with non-*P. falciparum *malaria, 64 patients with uncomplicated *P. falciparum *malaria and 14 patients with severe *P. falciparum *malaria).

**Methods:**

TREM-1, neopterin and procalcitonin were determined in serum using commercially available ELISA or EIA tests. The diagnostic performance of these biomarkers for severe disease was compared with plasma lactate, a well-validated parameter for disease severity in patients with malaria, as reference. Severe malaria was defined according to the modified WHO criteria.

**Results:**

No significant differences in TREM-1 levels were detected between the different patient groups. Patients with severe *P. falciparum *malaria had significantly higher neopterin and procalcitonin levels on admission when compared to patients with uncomplicated *P. falciparum *malaria or non-*P. falciparum *malaria. Receiver Operating Characteristic (ROC) curve analysis showed that neopterin had the highest Area-Under-the-ROC curve (AUROC 0.85) compared with plasma lactate (AUROC 0.80) and procalcitonin (AUROC 0.78). At a cut-off point of 10.0 ng/ml, neopterin had a positive and negative predictive value of 0.38 and 0.98 whereas procalcitonin, at a cut-off point of 0.9 ng/ml, had a positive and negative predictive value of 0.30 and 1.00.

**Conclusion:**

Although the diagnostic value of neopterin and procalcitonin is limited, the high negative predictive value of both neopterin and procalcitonin may be helpful for a rapid exclusion of severe malaria disease on admission. This may be a valuable tool for physicians only occasionally dealing with ill-returned travellers from malaria-endemic regions and who need to decide on subsequent oral anti-malarial treatment or timely referral to a specialized centre for high-level monitoring and intensified parenteral treatment.

## Background

Travellers from industrialized countries and inhabitants of malaria-endemic regions clearly represent two distinct worlds of malaria [1]. The global burden of malaria is largely carried by the world's malaria-endemic regions with as many as 500 million cases annually and a death toll of 1 to 3 million children each year. Severe malaria in areas of endemicity is associated with a mortality of 15 to 40% [2,3]. In many malaria-endemic regions, strict triage for admission to ICU facilities must be applied because the ICU capacity is usually limited. Recently, a 5-point Coma Acidosis Malaria (CAM) score based on only acidosis (base deficit) and cerebral malaria (measured with Glasgow Coma Scale) was introduced, which could identify adult patients with severe malaria who were at high risk of death [4].

In striking contrast, in non-endemic industrialized countries malaria is only seen as an occasionally imported disease [5] and is usually associated with a low case-fatality rate [6,7]. Even in the pre-artesunate era, the mortality of severe malaria in non-endemic regions was significantly lower when compared with regions of malaria endemicity [6-8], probably reflecting the availability of adequate supportive care facilities in industrialized countries.

Industrialized countries, however, have to face other -more trivial- problems. For instance, the expertise on diagnosis and treatment of malaria is usually focussed in some specialized hospitals and institutes but many ill-returning travellers may present to non-specialized hospitals or even general practitioners. Making a proper diagnosis of malaria may be troublesome under these circumstances, for instance, by lack of experience in the examination of malaria thick and thin blood smears and in the assessment of parasite load. These non-specialized centres therefore often rely on rapid diagnostic tests for the diagnosis of malaria [9]. Although sensitive in diagnosing *P. falciparum *malaria, these rapid tests do not provide any information about the severity of the infection. Moreover, although artesunate, which is now considered the parenteral drug of choice for treatment of severe falciparum malaria, is available as an orphan drug in The Netherlands, it is currently only in stock in some specialized centres but certainly not available in every Dutch hospital. Some of these general hospitals do not even have any drug in stock for the treatment of malaria [10]. To prevent unnecessary delay in diagnosis of severe malaria and institution of proper parenteral treatment, a simple, well-validated, laboratory-based biomarker that predicts or excludes severe disease accurately would be of great help for those clinicians occasionally dealing with febrile travellers returning from malaria endemic regions. These clinicians have to decide on subsequent oral anti-malarial treatment or a timely referral to a specialized centre for high-level monitoring and intensified parenteral treatment. In the present study, the diagnostic accuracy of plasma soluble Triggering Receptor Expressed on Myeloid cells 1 (TREM-1), neopterin and procalcitonin was evaluated as potential markers for malaria disease severity in travellers with imported malaria. These bio-substances are all involved in the systemic pro-inflammatory response of the host to invading pathogens. Some of these biomarkers are already in use for the diagnosis and follow-up of sepsis or used in treatment algorithms, resulting in a successful reduction of antibiotic use and duration [11,12].

## Methods

### Study population

The Harbour Hospital is a 161-bed general hospital located in Rotterdam. It also harbours the Institute for Tropical Diseases, which serves as a national reference centre. In the period 1999-2008 almost 500 cases of imported malaria were diagnosed [13]. For the majority of these cases, demographic, clinical and laboratory data and serum samples were available. For the present study, a representative sample of this cohort was taken and analysed.

### Definitions

Patients were classified as having severe *P. falciparum *malaria if they met one or more of the WHO criteria for severe malaria, as modified by Hien *et al *[14]:

• A score on the Glasgow Coma Scale of less then 11 (indicating cerebral malaria).

• Anaemia (haematocrit < 20%) with parasite counts exceeding 100,000/μl (roughly corresponding to 2% parasitaemia) on a peripheral blood smear.

• Jaundice (serum bilirubin > 50 μmol/l) with parasite counts exceeding 100.000/μl on a peripheral blood smear.

• Renal impairment (urine output < 400 ml/24 h and serum creatinine > 250 μmol/l).

• Hypoglycaemia (blood glucose < 2.2 mmol/l).

• Hyperparasitaemia (> 10% parasitaemia).

• Systolic blood pressure < 80 mm Hg with cold extremities (indicating shock).

### Study design

In previous studies [6,13,15] these severity criteria were also used to define severe malaria in non-immune travellers. In the present study the occurrence of severe malaria was considered a primary end-point. This contrasts with the design of many studies in patients with severe malaria in regions of malaria endemicity where the severity criteria are used as an entry criterion. In the present study, plasma lactate was used as a surrogate parameter for acid-base dysbalance and reference biomarker. It was evaluated in a previous study in non-immune travellers with imported malaria [15]. The diagnostic performance of TREM-1, procalcitonin and neopterin for malaria disease severity was compared with that of plasma lactate, which is routinely measured at the Institute for Tropical Diseases in ill-returning travellers.

### Procedures

On admission, blood samples were taken for analysis of the red blood cell count, haematocrit, white blood cell count, platelet count, serum electrolytes, total bilirubin, serum creatinine, liver enzymes, and blood glucose. In addition, a serum sample was taken on admission which was stored at -20°C until analysis. For the determination of plasma lactate, a separate blood sample was drawn on admission without congestion and placed on melting ice after which it was immediately analysed after isolation of plasma. Malaria was diagnosed by QBC (Quantitative Buffy Coat) analysis, by a rapid diagnostic antigen test for malaria (Binax NOW^® ^Malaria Test, Binax Inc., Maine, USA) and by conventional microscopy of stained thick and thin blood smears. In case of *P. falciparum *infections, parasite density was determined. When the parasitaemia was less than 0.5% infected erythrocytes, parasites were counted per 100 leucocytes in thick smears. When the parasitaemia was equal or higher than 0.5% infected erythrocytes, infected erythrocytes were counted in thin blood smear and expressed as a percentage of the total erythrocytes. The number of parasites per microliter was subsequently calculated from these data.

TREM-1 and neopterin levels were determined in serum samples using commercially available ELISA tests (R&D Systems, Abingdon, UK; DRG, Marburg, Germany, respectively). Procalcitonin levels in serum samples were determined using a commercially available EIA test (VIDAS BRAHMS Procalcitonin, bioMérieux, Lyon, France). All tests were performed according to manufacturer's instructions. Detection limits were 3.88 pg/ml for TREM-1, 0.2 ng/ml for neopterin and 0.05 ng/ml for procalcitonin, respectively. According to the manufacturers, normal serum values are < 100 pg/ml for TREM-1, < 3 ng/ml for neopterin and < 0.1 ng/ml for procalcitonin.

### Statistical methods

For comparison between groups, the Mann-Whitney U-test was used and p-values of < 0.05 were considered statistically significant. The diagnostic performance of each biomarker was reported as sensitivity, specificity, positive and negative predictive value for severe *P. falciparum *malaria and their corresponding 95% confidence intervals. Of each test a Receiver Operating Characteristic (ROC) curve, a graphical plot of sensitivity (true positive rate) versus 1-specificity (false positive rate), was constructed as a summary statistic and the area under the ROC curve (AUROC) and its corresponding 95% confidence intervals were calculated. Youden's index J (J = sensitivity+specificity-1) was used to choose the most appropriate cut-off point for each biomarker. All statistical analyses were performed using SPSS 15.0.

## Results

### Patient characteristics

In total 104 travellers with imported malaria were included in this study, of which 26 patients were diagnosed with a non-*P. falciparum *infection (*Plasmodium malariae *n = 2; *Plasmodium ovale *n = 5; *Plasmodium vivax *n = 19) and 78 patients were diagnosed with *P. falciparum *infection. The general characteristics of all patients are shown in Table 1.

**Table 1:** General characteristics and laboratory results on admission of patients with various species of malaria. Data are given as median (range).

	Non-*P. falciparum*	*P. falciparum*	
		Uncomplicated	Severe
	(n = 26)	(n = 64)	(n = 14)
**Demographics**			
Male/female	20/6	51/13	6/8
Age, years	40 (17-62)	40 (11-67)	40 (26-57)
**Continent of acquisition**			
Africa	12 (46%)	60 (94%)	12 (86%)
Asia	9 (35%)	3 (5%)	1 (7%)
South America	5 (19%)	1 (2%)	1 (7%)
**Vital signs on admission**			
Body temperature, °C	38.8 (36.1-41.5)	38.7 (36.1-40.6)	38.8 (36.8-40.6)
Pulse rate, beats per minute	90 (60-130)	95 (68-120)	108 (78-140)
Systolic blood pressure, mm Hg	123 (100-196)	120 (95-185)	118 (80-160)
**Laboratory data on admission**			
Parasite load, throphozoites/μl	ND	5,502 (1.0-385,000) *	205,600 (80,500-860,000)
Plasma lactate, mmol/l	1.4 (0.7-3.0) *	1.5 (0.5-4.4) *	2.6 (0.9-5.8)
Haemoglobin, mmol/l	8.2 (6.1-10.1)	8.7 (5.3-11.1) *	7.6 (3.8-10.2)
Leucocytes, × 10^9^/l	5.2 (1.9-9.3)	5.5 (1.8-11.3)	6.6 (3.2-18.5)
Platelets, × 10^9^/l	93.0 (10.0-205.0) *	78.5 (16.0-247.0) *	27.0 (3.0-152.0)
C-reactive protein, mg/l	86.5 (18.0-208.0) *	109.0 (5.0-278.0) *	190.0 (91.0-265.0)
Serum creatinine, μmol/l	94.0 (66.0-149.0)	103.5 (63.0-208.0)	102.5 (70.0-199.0)
Total bilirubin, μmol/l	24.0 (6.0-84.0) *	25.0 (7.0-164.0) *	54.0 (20.0-269.0)

p-values as compared with severe *P. falciparum *malaria.

* indicates p < 0.01

ND, not done

### Characteristics of patients with severe malaria

Thirteen patients fulfilled the criteria for severe malaria at initial presentation. Another patient did not fulfil these criteria on admission, but the clinical course deteriorated shortly hereafter with impaired consciousness and hyperparasitaemia. Procalcitonin and neopterin levels were already increased on admission in this particular patient. Eventually, at admission to the ICU, all 14 patients fulfilled one or more of the severity criteria (GCS < 11, n = 1; anaemia with a parasite count exceeding 100,000 trophozoites per μl, n = 2; icterus with a parasite count exceeding 100,000 trophozoites per μl, n = 8; acute oliguric renal insufficiency, n = 0; hypoglycaemia, n = 0; hyperparasitaemia, n = 5 and shock, n = 1, respectively). Five patients had an impaired conscious level but a GSC above 11; eight patients had a parasitaemia > 5%, respectively. The first arterial blood gas analysis on ICU showed a median bicarbonate level of 22 mmol/l (range 17 to 26 mmol/l) and a median base deficit of 2 (range -3 to 8). Median GCS was 15 (range 9 to 15). One patient needed mechanical ventilation. Eleven patients received exchange transfusion as an adjunct therapy. No case fatalities were observed. The laboratory results on admission of travellers with imported severe *P. falciparum *malaria were further characterized by significantly lower platelet counts and haemoglobin levels and by significantly higher plasma lactate, bilirubin and C-reactive protein levels and erythrocyte sedimentation rates, respectively (Table 1).

### Analysis of biomarkers for severe malaria

#### TREM-1

**Figure 1:**
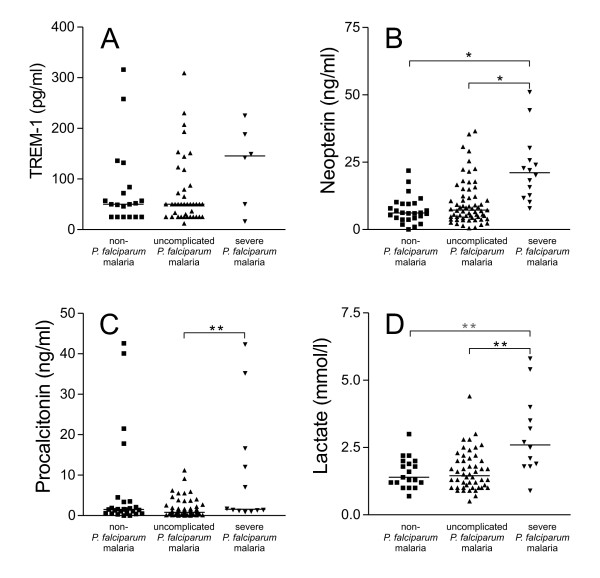
**Concentrations of potential biomarkers for disease severity in malaria patients on admission**. Individual data are shown with the median value of each biomarker; TREM-1 (panel A), neopterin (panel B), procalcitonin (panel C) and plasma lactate (panel D). Significant differences in biomarker concentrations between patient groups (black square = non-*P. falciparum *malaria; black triangle up = uncomplicated *P. falciparum *malaria; black triangle down = severe *P. falciparum *malaria) with P values < 0.0001 and < 0.005 are indicated by * and **, respectively.

No statistically significant differences were observed in TREM-1 levels in serum, between patients with severe *P. falciparum *malaria, uncomplicated *P. falciparum *malaria and non-*P. falciparum *malaria (Figure 1A).

#### Neopterin

**Figure 2:**
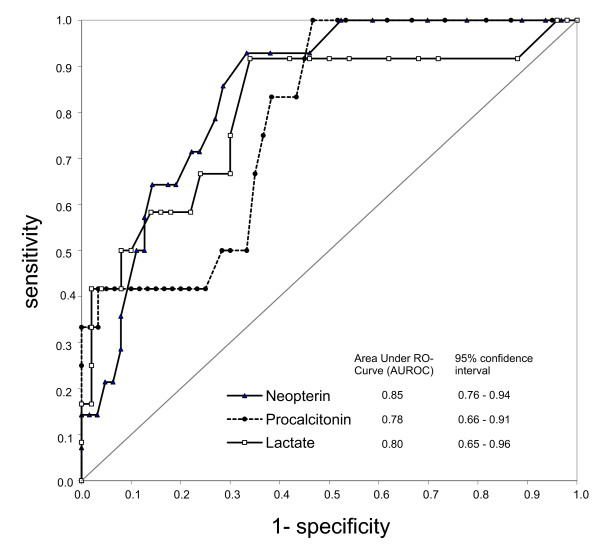
**Receiver Operating Curves (ROC) characteristics of the diagnostic performance of neopterin, procalcitonin and lactate for severe *P. falciparum *malaria**. The ROC curve is a graph of sensitivity (true positive fraction) plotted against 1-specificity (false positive fraction). The performance of a diagnostic variable can be quantified by calculating the area under the ROC curve (AUROC). The ideal test would have an AUROC of 1, whereas a random guess would have an AUROC of 0.5.

Neopterin levels on admission were significantly higher in travellers with severe *P. falciparum *malaria when compared to travellers with uncomplicated *P. falciparum *malaria (p < 0.0001) and travellers with non-*P. falciparum *malaria (p < 0.0001) (Figure 1B). ROC curve analysis showed an AUROC of 0.85 (95% Confidence Interval 0.76-0.94), suggesting a good accuracy (Figure 2). As shown in Table 2, at a cut-off point of 10.0 ng/ml, neopterin had an excellent sensitivity and negative predictive value but a poor specificity and positive predictive value for severe disease.

**Table 2:** Descriptive statistics of diagnostic accurary of neopterin, procalcitonin as compared with lactate for the diagnosis of severe *falciparum *malaria on admission.

	Neopterin 95% Confidence Interval		Procalcitonin 95% Confidence Interval		Lactate 95% Confidence Interval	
**Optimal cut-off value**	10 ng/ml		0.9 ng/ml		1.8 mmol/l	
**Youden's index**	0.60		0.53		0.58	
**Sensitivity**	0.93	0.64-1.00	1.00	0.70-1.00	0.92	0.60-1.00
**Specificity**	0.67	0.54-0.78	0.53	0.40-0.66	0.66	0.51-0.78
**Positive predictive value**	0.38	0.23-0.56	0.30	0.17-0.47	0.39	0.22-0.59
**Negative predictive value**	0.98	0.86-1.00	1.00	0.87-1.00	0.97	0.83-1.00

The Youden's index was used to choose an appropriate cut-off value.

#### Procalcitonin

Procalcitonin levels were significantly higher in travellers with severe *P. falciparum *malaria when compared to travellers with uncomplicated *P. falciparum *malaria (p = 0.0022). However, no significant differences were noted in comparison to travellers with non-*P. falciparum *infections (p = 0.17) (Figure 1C). ROC curve analysis showed an AUROC of 0.78 (95% CI 0.66-0.91), compatible with a fair accuracy (Figure 2). At a cut-off point of 0.9 ng/ml, procalcitonin had an excellent sensitivity and negative predictive value, whereas specificity and positive predictive value for severe *P. falciparum *malaria was poor (Table 2).

#### Plasma lactate

Plasma lactate levels were significantly higher in travellers with severe *P. falciparum *malaria when compared to travellers with uncomplicated *P. falciparum *malaria (p = 0.0012) and travellers with non-*P. falciparum *malaria (p = 0.0040). ROC curve analysis of plasma lactate levels showed an AUROC of 0.80 (95% CI 0.65-0.96) compatible with a good accuracy (Figure 2). At a cut-off point of 1.8 mmol/l, lactate had an excellent sensitivity and negative predictive value, but a poor specificity and positive predictive value for severe *P. falciparum *malaria (Table 2), respectively.

#### Combination of various biomarkers for severe falciparum disease

Analysis of various combinations of newly tested biomarkers and the use of different cut-off levels did not result in better discrimination of patients with severe *P. falciparum *malaria.

## Discussion

Severe malaria is disreputable for its high case-fatality rate, but the outcome of severe *P. falciparum *infections has significantly improved since the introduction of artesunate as first line treatment of severe malaria, in particular in developing countries [2]. In industrialized countries such as The Netherlands, the case-fatality rate of imported malaria is low and fatal cases are only occasionally reported. In the present study, in which the biomarkers TREM-1, neopterin and procalcitonin were evaluated for their potential to be used as a marker for severe malaria disease upon admission. This contrasts with the design of many studies in regions of malaria endemicity where severe malaria is usually the entry criterion. For reasons of comparability, the same set of criteria for severe malaria was strictly applied for the diagnosis of severe malaria in this study, even though the study population comprised of presumably non-immune travellers and some authors even suggest a threshold of 5% in stead of 10% parasitized erythrocytes to define hyperparasitaemia in non-immune individuals.

The quantification of soluble TREM-1 levels on admission did not result in proper discrimination of severe *P. falciparum *malaria from uncomplicated *P. falciparum *malaria and non-*P. falciparum *malaria. In contrast, travellers with severe *P. falciparum *malaria had significantly higher levels of neopterin and procalcitonin on admission as compared with travellers with uncomplicated *P. falciparum *malaria or non-*P. falciparum *malaria, respectively. These findings correspond with the results of several other studies performed in semi-immune malaria patients living in malaria-endemic regions [16-18]. When the ROC curve characteristics of neopterin and procalcitonin were compared to that of plasma lactate, the AUROC of neopterin appeared superior whereas the AUROC of procalcitonin appeared inferior to that of lactate, suggesting that neopterin provided the most accurate diagnostic performance for severe *P. falciparum *malaria in this cohort of travellers.

Unfortunately, the applicability of these tests in the initial clinical assessment of patients with severe *P. falciparum *malaria will probably be limited by the poor positive predictive value of neopterin and procalcitonin indicating that neither test can serve as a valuable tool for the diagnosis of severe *P. falciparum *malaria. For illustration, applying a procalcitonin level > 0.9 ng/ml or a neopterin level > 10.0 ng/ml as a guide to intensified monitoring and treatment would result in more than 20 of 64 patients with uncomplicated *P. falciparum *malaria receiving more intensive monitoring and treatment than strictly necessary. On the other hand, the high negative predictive value of both neopterin and procalcitonin suggests that these tests can still be of value by providing a tool for exclusion of severe disease. With either a procalcitonin level of less than 0.9 ng/ml or a neopterin level of less than 7.9 ng/ml in serum on admission as a cut-off point for severe *P. falciparum *malaria, no patient with severe disease would have been denied access to high-level monitoring and intensive treatment. In a previous study, in which a semi-quantitative 'point-of-care' procalcitonin test as a diagnostic tool for severe *P. falciparum *malaria was evaluated prospectively, all 6 patients with severe *P. falciparum *malaria had procalcitonin values classified as either "moderate" or "high" (corresponding to a procalcitonin level ≥ 2 ng/ml), but never as "normal" or "low" [12]. This is compatible with the findings of the current retrospective serum sample-based study in which procalcitonin was measured quantitatively.

Although severe or fatal malaria rarely results from infections with the non-sequestering *Plasmodium *species *vivax*, *ovale *and *malariae*, increased neopterin and procalcitonin serum levels were also observed in the majority of these patients, although levels were lower than compared with severe *P. falciparum *malaria patients. Although speculatively, these observations suggest that the mechanism whereby neopterin and procalcitonin levels increase in malaria, is not specific for severe *P. falciparum *malaria alone. Therefore, it may not accurately reflect the pivotal pathophysiological events in complicated *P. falciparum *malaria, such as the sequestration of infected red blood cells in the microcirculation of vital organs and disturbance of microcirculatory flow. Whereas an increased plasma lactate level conceivably reflects a significant reduction in microcirculatory flow in vital organs, the elevated neopterin and procalcitonin levels are probably the result of activation of a common inflammatory host response evoked by infection with the respective *Plasmodium *parasites. In fact, some reports even suggest that *P. falciparum *malaria per se is not associated with a stronger host response than *P. vivax *or *P. ovale *malaria, but that the parasite burden of the causative *Plasmodium *species may also modulate the extent of the host inflammatory response [19].

In conclusion, although neither neopterin nor procalcitonin can probably serve as a useful single diagnostic tool for severe *P. falciparum *malaria, the high negative predictive value of both neopterin and procalcitonin may be helpful for a rapid exclusion of severe *P. falciparum *malaria on admission. This may be a valuable tool - particularly if available as a rapid diagnostic test - for physicians only occasionally dealing with ill-returned travellers and who need to decide on subsequent oral anti-malarial treatment or a timely referral to a specialized centre for high-level monitoring and intensified parenteral treatment.

## Competing interests

The authors declare that they have no competing interests.

## Authors' contributions

RW participated in the design of the study and coordination, performed the experiments and the statistical analyses and drafted the manuscript.

MW participated in the statistical analyses and drafting of the manuscript.

PP participated in the design of the study.

JH participated in the design of the study and revising the manuscript.

RK is responsible for collection of patient materials and database management.

AB participated in the design of the study and revising the manuscript.

PG participated in the design and coordination of the study and in drafting and revising the manuscript.

All authors have seen and approved the final version.
